# Primary motor cortex deactivation as a new mechanism of motor inhibition in conversion paralysis

**DOI:** 10.1002/mds.27552

**Published:** 2018-11-28

**Authors:** Eva Matt, Ahmad Amini, Tuna Aslan, Robert Schmidhammer, Roland Beisteiner

**Affiliations:** ^1^ Department of Neurology Medical University of Vienna Vienna Austria; ^2^ High Field Magnetic Resonance Centre Medical University of Vienna Vienna Austria; ^3^ Ludwig Boltzmann Institute for Experimental and Clinical Traumatology Vienna Austria

Conversion disorder (CD) patients may show a highly impressive picture of complete paralysis without an identifiable organic cause. The decisive mechanism of how specific motor functions are suppressed in these patients is still under debate. Here we provide a comprehensive clinical and functional imaging investigation of a female patient (aged 38 years) who experienced a direct trauma of her left shoulder that was followed by anesthesia and paralysis of the left arm. She underwent detailed clinical examinations, structural MRI, ultrasonography, electromyography, nerve conduction velocity, EEG, motor evoked potentials (Fig. 1A), median nerve somatosensory evoked potentials, and microsurgical inspection of the brachial plexus including intraoperative electrical nerve stimulation. As no neurological findings could explain the patient's persisting symptoms, she was diagnosed with CD.

**Figure 1:**
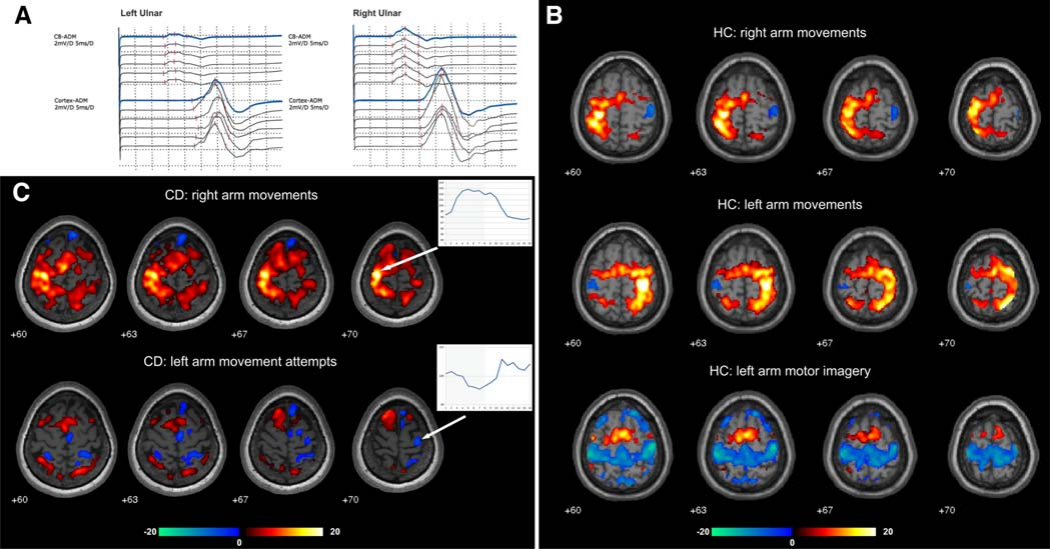
(**A**) Motor evoked potentials in the conversion disorder (CD) patient for the paralyzed left and the healthy right ulnar nerve (recording site: abductor digiti minimi muscle [ADM]). The motor evoked potential latency and amplitude were within normal ranges without significant differences between both sides. (**B**) Task‐related activations (red–yellow) and deactivations (green–blue) in the healthy controls (HC) while performing normal arm movements and motor imagery. During normal motor execution deactivations were restricted to the primary motor cortex ipsilateral to the movement while during motor imagery widespread deactivations in the bilateral primary sensorimotor cortex were found. (**C**) Task‐related activations and deactivations in the CD patient and mean time course averaged over all blocks and runs (activation period indicated by gray background color) calculated for a 4 mm sphere centered on the activation peak (right arm movements) and deactivation peak (left arm movements) in the primary motor cortex in the CD patient. Movements of the healthy right arm elicited strong activations in the respective primary motor cortex with a signal increase during movement blocks (indicated by gray background color) for the activation peak (arrow). During movement attempts of the paralyzed left arm, the patient showed deactivation of the respective primary motor cortex further illustrated as signal drop during the motor task. [Color figure can be viewed at wileyonlinelibrary.com]

During functional magnetic resonance imaging (fMRI) the patient performed movements with the healthy arm and movement attempts with the paralyzed arm (alternated with rest periods). Three healthy controls performed either normal execution or motor imagery of arm movements using a “conversion like” instruction (“vividly imagine but not execute”). As expected, normal movements in the controls resulted in a strong activation in the respective contralateral sensorimotor cortex and premotor areas (Fig. 1B). When moving the healthy arm, the CD patient showed similar activation patterns as the controls, but during her attempts to move the paralyzed arm M1 activations were completely missing (Fig. 1C). Instead, the patient displayed deactivations in the motor representation of the left arm. Although previous studies report lower M1 activation in CD patients with limb paresis or paralysis when compared with the heathy side,^1^, ^2^, ^3^ this is the first report of M1 deactivation, that is, a signal decrease during the attempt to move relative to a resting phase. A deactivation or a negative blood‐oxygenation‐level‐dependent response reflects an attenuation of the local blood flow that most likely is caused by suppressed or reduced neuronal activity.^4^

Surprisingly, expanded M1 deactivations were also found in the controls during motor imagery (Fig. 1B), potentially as a mechanism to prevent movement execution as imposed by the “conversion like” instruction. A systematic review reported that approximately 82% of motor imagery experiments found a lack of activation in M1, but none of these studies reported deactivations.^5^ A probable reason for this is that the vast majority of fMRI studies solely focus on activations. It seems likely that extending classical fMRI analyses to task‐related deactivations will increase evidence for this correlate of motor inhibition. Regarding the activations during motor imagery, our results of the recruitment of premotor areas and of the fronto‐parietal control network are consistent with previous reports.^5^ In contrast, the CD patient showed an increased prefrontal recruitment during the movement attempts, supporting the hypotheses that hyperactive emotional processes interfere with motor functions.^6^, ^7^ Although mediated by differential networks, both active control (motor imagery in the controls) and involuntary suppression (conversion paralysis) of movement attempts can lead to deactivation of M1 causing movement inhibition. Considering not only functional activations but also task‐related deactivations will further our understanding of motor inhibition under physiological and pathophysiological conditions.

## Author Roles

1) Research project: A. Conception, B. Organization, C. Execution; 2) Statistical analysis: A. Design, B. Execution, C. Review and Critique; 3) Manuscript preparation: A. Writing of the first draft, B. Review and Critique.

E.M.: 1A, 1B, 1C, 2A, 2B, 3A

A.A.: 1B, 1C, 2C, 3B

T.A.: 1B, 1C, 2C, 3B

R.S.: 1A, 1B, 2C, 3B

R.B.: 1A, 1B, 1C, 2C, 3A, 3B

## Financial Disclosures (for the preceding 12 months)

E.M. reports grants from the Austrian Federal Ministry of Education, Science and Research, Austrian Research Association (International Communication). A.A. reports grants from the Austrian Federal Ministry of Education, Science and Research. T.A. reports grants from the Austrian Science Fund. R.S. reports consultancies. R.B. reports grants from the Austrian Science Fund, Austrian Federal Ministry of Education, Science and Research, and Medical University of Vienna.
